# The adenovirus E1A oncoprotein N-terminal transcriptional repression domain enhances p300 autoacetylation and inhibits histone H3 Lys18 acetylation

**DOI:** 10.18632/genesandcancer.47

**Published:** 2015-01

**Authors:** Ling-Jun Zhao, Paul M. Loewenstein, Maurice Green

**Affiliations:** ^1^ Institute for Molecular Virology, Saint Louis University School of Medicine, Doisy research Center, St. Louis, Missouri

**Keywords:** E1A 1-80, H3K18 acetylation, reconstituted chromatin, p300, autoacetylation

## Abstract

Expression of the adenovirus E1A N-terminal transcription repression domain alone (E1A 1-80) represses transcription from specific promoters such as HER2 [1] and from reconstituted chromatin [2]. Significantly, E1A 1-80 can induce the death of human breast cancer cells over-expressing the HER2 oncogene [1] as well as other cancer cells. Here, we report that E1A 1-80 alone is sufficient to inhibit H3K18 acetylation in vivo and p300-mediated H3K18 acetylation in reconstituted chromatin. Of interest, hypoacetylation of H3K18 has been correlated with the survival of tumor cells and the poor prognosis of many cancers [3, 4]. E1A 1-80 enhances p300 autoacetylation and concurrently inhibits H3K18 acetylation in chromatin in a dose-dependent manner. Pre-acetylation of p300 by incubation with acetyl-CoA alone reduces p300's ability to acetylate H3K18 in chromatin. Additional acetylation of p300 in the presence of E1A 1-80 produces stronger inhibition of H3K18 acetylation. These findings indicate that autoacetylation of p300 greatly reduces its ability to acetylate H3K18. The results reported here combined with our previous findings suggest that E1A can repress transcription by multiple strategies, including altering the chromatin modifying activity of p300 and dissociating TFIID from the TATA box thus disrupting formation of the transcription pre-initiation complex [5, 6]

## INTRODUCTION

The first gene expressed after adenovirus (Ad) infection is early gene 1A (E1A). Group C Ad E1A transcribes two highly related mRNAs which code for two regulatory proteins (E1A 243R and E1A 289R). Discrete domains of E1A interact with key regulatory proteins and confer diverse functions including transcriptional activation, transcriptional repression, induction of cellular DNA synthesis, cell immortalization, cell transformation, as well as inhibition of metastasis and cell differentiation [7-11]. E1A 289R differs from E1A 243R by conserved region 3 (CR3) (amino acid residues 140-185), a 46 amino acid domain unique to 289R. CR3 is essential and sufficient for transcriptional activation of Ad early genes [12, 13].

An important function encoded in the E1A 243R protein N-terminus is transcriptional repression of cellular genes involved in cell proliferation and differentiation [14, 15]. We have demonstrated that the transcriptional repression function of the E1A oncogene resides solely within the N-terminal 80 amino acids of E1A (E1A 1-80) [16, 17], and that E1A 1-80 exhibits the same repression function as the entire E1A 243R protein. Single amino acid substitution analysis showed that there are two E1A N-terminal repression sub-domains which interact with the cellular multifunctional histone acetyl transferase (HAT), p300, as well as the basal transcription machinery through TBP (TATA-binding protein). These studies suggested a two-step model of E1A repression [16, 18]: first, E1A gains access to repressible promoters by interaction of E1A repression sub-domain 1 (amino acids ~1-30) and 2 (amino acids ~48-60) with a promoter-bound cellular target such as p300; second, the E1A N-terminus sub-domain 1 then interacts with TFIID and disrupts the TFIID/TATA complex thus blocking pre-initiation complex (PIC) formation [6, 18].

E1A 1-80 can repress some promoters of clinical significance such as the HER2 proto-oncogene [1]. Of significance, when E1A 1-80 is expressed in human breast cancer cells that over-express HER2, not only is HER2 expression repressed but HER2 over-expressing cancer cells die. In contrast, normal cells are not killed by E1A 1-80. Since chromatin is the natural template for transcriptional regulation, we have recently examined E1A-mediated transcriptional repression in reconstituted chromatin, and found that transcription from an E1A-repressible template in chromatin is effectively repressed by E1A 1-80 and E1A 243R [2].

A major target of the E1A repression function, p300 and its close homolog CBP are co-activators targeted by many transcription factors involved in regulation of many cellular genes that function in various metabolic pathways [19-21]. Spontaneous mutations of p300/CBP and targeted inhibition of p300 expression can have either pro- or anti-tumor functions, depending on the types of tumors involved [21, 22]. Thus, p300 HAT activity plays an important role not only in gene regulation but also in cell proliferation and death. The main substrates for the p300 HAT enzyme are histones H3 and H4, with H3K4, H3K18, H4K5 and H4K8 being the most favored residues [23]. Significantly, p300 appears to be the key HAT involved in global cellular acetylation of H3K18, and hypoacetylation of H3K18 is correlated with poor prognosis of cancers [4, 24]. Importantly, p300 autoacetylates and autoacetylation appears to be involved in regulation of p300 structure and activity [25], as well as p300 release from the promotor during pre-initation complex PIC formation [26]. In this report, we investigate the effect of the E1A N-terminal repression domain on p300 autoacetylation and on p300-mediated H3K18 acetylation in the context of in vitro assembled chromatin.

## RESULTS

### E1A 1-80 inhibits H3K18 acetylation in vivo and in vitro

**Figure 1:**
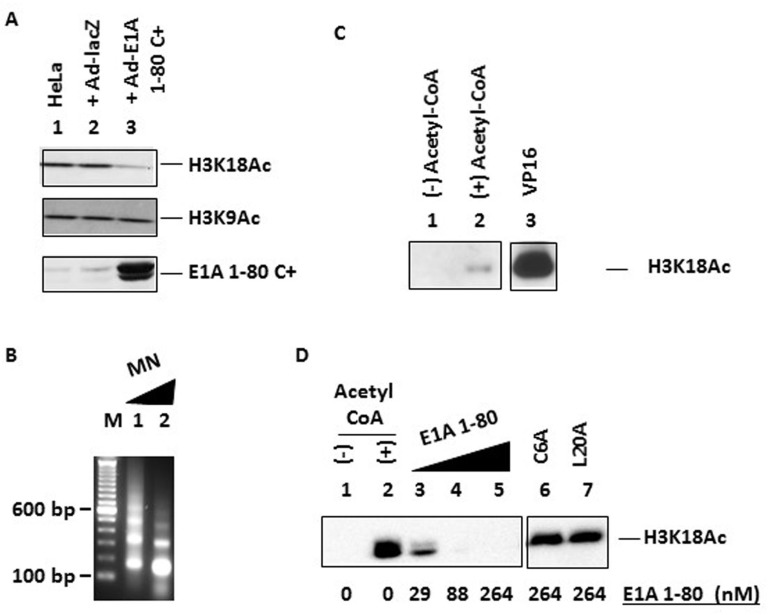
Inhibition of H3K18 acetylation by E1A 1-80 in vivo and in vitro **A.** Inhibition of H3K18 acetylation, but not H3K9 acetylation, by E1A 1-80 C+ in vivo. HeLa cells were infected with Ad-lacZ or Ad-E1A 1-80 C+ and lysed for Western blot analyses with the indicated antibodies. Top panel: H3K18Ac antibody; middle panel: H3K9Ac antibody; bottom panel: rabbit polyclonal antibody against E1A 1-80. **B.** Analysis of in vitro assembled chromatin by micrococcal nuclease (MN) digestion. Chromatin was digested with MN at room temperature for 0.5 min (lane 1) or 3 min (lane 2). M: 100-bp DNA ladder. **C.** In vitro histone acetylation reactions with reconstituted chromatin. All lanes contained p300 and assembled chromatin. Gal4-VP16 (VP16) was used for reaction in lane 3 only. Reaction products were subjected to Western blot analyses with antibody against H3K18Ac, and the X-ray film image is shown. Lane 3 was cropped from the same image. **D.** Inhibition of H3K18 acetylation by E1A 1-80, but not by the C6A and L20A mutants. Conditions were the same as in C, except that all lanes contained Gal4-VP16 and Western blot was developed by chemiluminescence and visualized by directly on a Bio-Rad scanner. Lane 1: without acetyl-CoA as a negative control. Lanes 6 & 7 were cropped from the same image.

It has been reported that Ad E1A 243R globally inhibits p300-mediated acetylation of histone H3K18 in vivo, but has no effect on acetylation of H3K9 [4]. Since we have shown that the Ad E1A N-terminal repression domain, E1A 1-80, targets p300 and represses transcription [27], we asked whether the ability to inhibit H3K18 acetylation is encoded within E1A 1-80. HeLa cells were infected with an Ad vector expressing E1A 1-80 C+, which carries a V5 epitope tag at the C-terminus of E1A 1-80 and exhibits strong transcriptional repression and cancer cell killing activities [1]. Cell lysates were examined for the levels of H3K18Ac and H3K9Ac by Western blot analysis with acetylation-specific antibodies (see “Materials and Methods”). Expression of E1A 1-80 C+ resulted in inhibition of H3K18 acetylation (Figure 1A, top panel, lane 3) but not that of H3K9 (middle panel), whereas expression of control lacZ had no effect (lane 2). E1A 1-80 C+ was expressed as expected (lane 3, bottom panel).

Since transcription and histone acetylation occur in the context of chromatin, we examined the effect of E1A 1-80 on histone acetylation in reconstituted chromatin. Chromatin was assembled using purified core histone, ATP-dependent chromatin assembly factors, and the transcription template pG5MLT [2, 28]. The E1A 1-80-repressible pG5MLT template contains the core Ad ML (Major Late) promoter downstream of five copies of the Gal4-binding site that allow transcriptional activation by Gal4-VP16. When reconstituted chromatin was partially digested with micrococcal nuclease, the expected nucleosomal DNA ladder was observed (Figure 1B). When reconstituted chromatin was used for in vitro acetylation assays, H3K18 was acetylated by p300 to only a low level (Figure 1C, lane 2). This result indicates that H3K18 within non-activated reconstituted chromatin is not efficiently acetylated by p300.

Transcriptional activators, through binding to either enhancers or promoter-proximal regions, can recruit histone modifiers and chromatin remodelers to induce changes in the nucleosome occupancy/positioning around the promoter, and consequently, changes in gene expression [29, 30]. Therefore, purified Gal4-VP16 activator, which binds to the Gal4-binding sites in the pG5MLT template, was added to the acetylation reaction with p300 and reconstituted chromatin. In the presence of Gal4-VP16, p300 strongly acetylated H3K18 (Figure 1C, lane 3).

Using conditions of strong H3K18 acetylation when both p300 and Gal4-VP16 were present, we examined the effects of increasing amounts of purified E1A 1-80, and two mutants, C6A and L20A, that are defective in transcriptional repression and do not effectively interact with p300 [18]. As shown (Figure 1D), E1A 1-80 strongly inhibited H3K18 acetylation (lanes 3-5), whereas C6A (lane 6) and L20A (lane 7) were both defective. Under these conditions, acetylation of H3K9 was not detected (data not shown), which is likely due to the fact that H3K9 is not a favored substrate for p300 in vitro [23]. Because H3K18 is one of the four lysine residues favored by p300 in vitro [23] and its acetylation status appears to be correlated with cell transformation [3, 4], we further focused on E1A 1-80-mediated inhibition of H3K18 acetylation by p300.

### E1A 1-80 enhances p300 autoacetylation and inhibits H3K18 acetylation in a dose-dependent manner

E1A 1-80 inhibition of H3K18 acetylation by p300 could simply be due to competition between E1A 1-80 and histone H3 for access to p300. Alternatively, E1A 1-80 interaction with p300 may alter its conformation and reduce its catalytic activity towards histone H3 in chromatin. p300 has been shown to autoacetylate, which may impact p300 structure and function and its targeting of the preinitiation complex (PIC) [25, 26]. This could also alter its ability to acetylate H3K18. Because E1A 1-80 targets p300 for transcriptional repression [16, 18, 27], we initially examined the possibility that E1A 1-80 affects the autoacetylation of p300.

**Figure 2:**
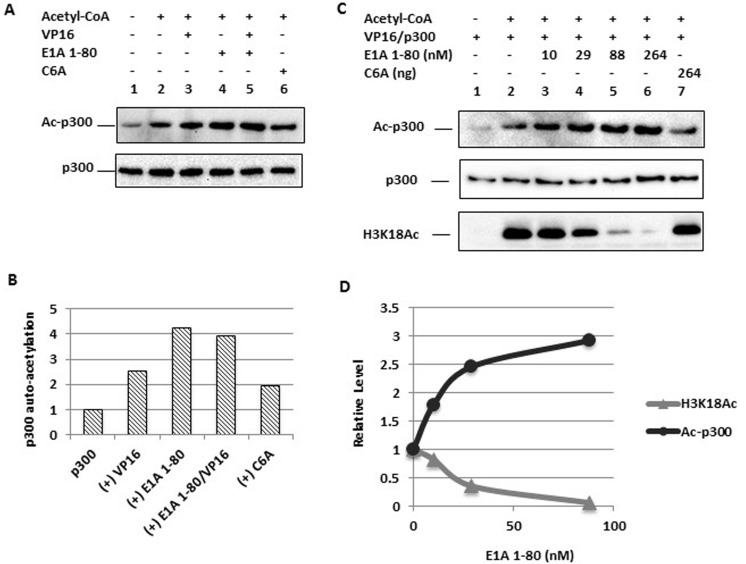
Enhancement of p300 autoacetylation by E1A 1-80 and correlation with inhibition of H3K18 acetylation **A.** p300 autoacetylation is enhanced by E1A 1-80. In vitro acetylation assay was performed with p300, VP16 (Gal4-VP16) (lanes 3 and 5), E1A 1-80 (264 nM, lanes 4 and 5), or C6A mutant (lane 6) in the absence of chromatin. Western blots were performed with acetyl-Lys antibody (for acetylated p300, Ac-p300, upper panel) or p300 antibody (lower panel). **B.** Quantitation of autoacetylated p300. Western blot chemiluminescence was quantitated on a Bio-Rad scanner directly (see “Materials and methods”), and normalized to the Ac-p300 level of lane 2. Data from lanes 2-6 were plotted. **C.** Titration of E1A 1-80 for its modulation of p300 autoacetylation and H3K18 acetylation. In vitro acetylation reaction was performed with p300 and VP16 (Gal4-VP16) with assembled chromatin, and varying amounts of E1A 1-80 (lanes 3-6), or C6A mutant (lane 7). Western blots were performed with antibodies as indicated. **D.** Quantitation of p300 autoacetylation and H3K18 acetylation. Quantitation of Western blot signals was as in B. Data from lanes 2-5 were plotted.

In vitro acetylation reactions were performed with p300, Gal4-VP16, E1A 1-80 and its C6A mutant in different combinations in the absence of chromatin (Figure 2A). Reaction products were examined for acetylated p300 (Ac-p300) by Western blot analysis with an antibody against acetylated lysine. E1A 1-80 significantly enhanced p300 autoacetylation in the absence of Gal4-VP16 (Figure 2A, upper panel, lane 4,) or in the presence of Gal4-VP16 (upper panel, lane 5) (see Figure 2B for quantitation). In contrast, the C6A mutant was substantially defective in enhancement of p300 autoacetylation (upper panel, lane 6). p300 levels were constant among the different assays as measured by Western blot analysis (Figure 2A, lower panel).

We next determined whether p300 autoacetylation and inhibition of H3K18 acetylation in chromatin are intrinsic properties of the E1A repression domain. A dose-response analysis with E1A 1-80 during in vitro acetylation of p300 was performed with reconstituted chromatin. Increasing amounts of E1A 1-80 resulted in increased levels of p300 autoacetylation (Figure 2C, upper panel), whereas acetylation of H3K18 was reduced in a dramatic fashion (Figure 2C, lower panel). Inhibition of H3K18 acetylation by E1A 1-80 was detected at a roughly equamolar ratio of E1A 1-80/p300 (10 nM E1A 1-80 vs 12 nM of p300) (Figure 2C, bottom panel, lane 3). At 88 nM of E1A 1-80 (lane 5), inhibition of H3K18 acetylation was almost complete. The C6A mutant was defective in inhibition of H3K18 acetylation (Figure 2C, bottom panel, lane 7). These data, as illustrated in Figure 2D, suggest that E1A 1-80 enhancement of p300 autoacetylation correlates with the inhibition of H3K18 acetylation.

### Time-dependent p300 autoacetylation in the presence or absence of E1A 1-80

**Figure 3:**
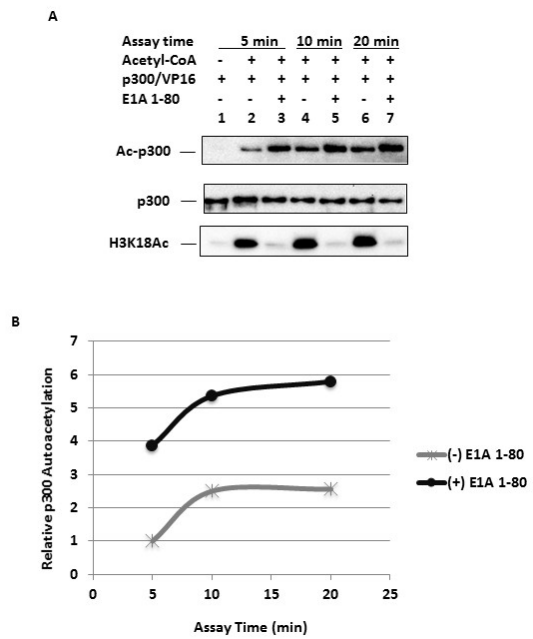
Time-dependent changes of p300 autoacetylation in the presence and absence of E1A 1-80 **A.** p300 autoacetylation at different times of reaction. In vitro acetylation reactions were performed with p300, VP16 (Gal4-VP16), and assembled chromatin, in the presence or absence of E1A 1-80 (264 nM). Assays were performed at 320C for 5, 10 or 20 minutes as indicated. Western blots were performed with antibodies as indicated. Acetylated p300 (Ac-p300) was detected with the Ac-Lys antibody (top panel). **B.** Quantitation of autoacetylated p300. Western blot signals from A were quantitated by chemiluminescence directly on a Bio-Rad scanner. H3K18 acetylation reached near maximum at 5 min of reaction and inhibition by E1A 1-80 was greater than 97% for all three time points (not shown).

To examine the progress of p300 autoacetylation and the effect of E1A 1-80 with reconstituted chromatin, acetylation reactions were performed in the presence or absence of E1A 1-80, and analyzed at different time points for both p300 autoacetylation and H3K18 acetylation. p300 autoacetylation was detected at 5 min of incubation (Figure 3A, top panel, lane 2) and was strongly enhanced by E1A 1-80 (lane 3). At 10 min of incubation, p300 autoacetylation approached completion (lane 4), as little change in p300 autoacetylation was observed at 20 min (lane 6). At each time, E1A 1-80 enhanced p300 autoacetylation (lanes 3, 5, 7). Similarly, H3K18 acetylation approached completion at 10 minutes of incubation (bottom panel, lane 4). As expected, E1A 1-80 inhibited H3K18 acetylation at each time point examined (bottom panel, lanes 3, 5, 7). Quantitative analysis of p300 autoacetylation indicated that E1A 1-80 enhancement of p300 autoacetylation ranged from 2-fold to about 4-fold (Figure 3B). Since the saturation level of p300 autoacetylation is different in the presence or absence of E1A 1-80, E1A 1-80 may enhance p300 autoacetylation at lysine residues that are normally not acetylated or only acetylated inefficiently by p300 alone.

### p300 autoacetylation inhibits chromatin H3K18 acetylation

**Figure 4:**
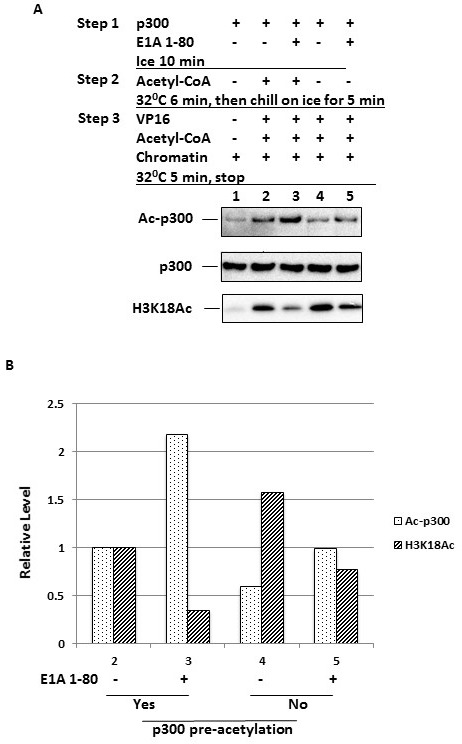
Increased p300 autoacetylation results in decreased H3K18 acetylation **A.** Pre-autoacetylation of p300 inhibits H3K18 acetylation. In vitro acetylation reactions were performed in a 3-step protocol as illustrated. p300 was first incubated with E1A 1-80 (29 nM) on ice (Step 1), and then acetyl-CoA was added to one set of reactions to initiate p300 autoacetylation (Step 2). Reactions were stopped by incubation on ice, and then other components added as indicated and H3K18 acetylation initiated (Step 3). Reaction products were examined by Western blot analysis. Acetylated p300 (Ac-p300) was detected with the Ac-Lys antibody (top panel). **B.** Quantitation of p300 autoacetylation and H3K18 acetylation. Values on X-axis correspond to the lane numbers in A.

Since E1A 1-80 concurrently enhances p300 autoacetylation and inhibits H3K18 acetylation, it is possible that p300 autoacetylation reduces its ability to acetylate H3K18 in chromatin. If this is the case, then allowing p300 to autoacetylate in the absence of chromatin should render p300 less capable of acetylating H3K18 when chromatin is subsequently provided. To examine this possibility, p300 was incubated on ice with (Figure 4A, lanes 3 and 5) or without E1A 1-80 (lanes 2 and 4) in duplicate sets (step 1). To observe E1A 1-80 effects more quantitatively, a limiting concentration of E1A 1-80 (29 nM) was used. Acetyl-CoA was then added to one set of the assays (lanes 2 and 3) to initiate pre-acetylation of p300 (step 2). After stopping pre-acetylation of p300 on ice for 5 min, Gal4-VP16, chromatin, and fresh acetyl-CoA were added to both sets of reactions, thus allowing acetylation of H3K18 to proceed (step 3). As shown in Figure 4A, pre-acetylation of p300 resulted in an autoacetylated p300 (top panel, compare lanes 2 and 3 with lanes 4 and 5). The amount of autoacetylated p300 was highest when p300 was pre-acetylated in the presence of E1A 1-80 (lane 3). Significantly, the level of acetylated H3K18 was lowest in this reaction (bottom panel, lane 3), consistent with the hypothesis that p300 autoacetylation reduces its ability to acetylate H3K18. Even in the absence of E1A 1-80, p300 pre-acetylation was correlated with a lower level of H3K18 acetylation (bottom panel, compare lane 2 with lane 4). When p300 was pre-acetylated, E1A 1-80 appears to inhibit H3K18 acetylation more efficiently (bottom panel, compare lanes 3 and 5). The quantitation of these results is presented in Figure 4B. As shown, compared to the absence of p300 pre-autoacetylation in lanes 4 and 5, data for lane 3 indicate that p300 pre-autoacetylation in the presence of E1A 1-80 results in the largest inhibition of H3K18 acetylation.

## DISCUSSION

p300 is recruited by many transcription factors as a coactivator and functions as a master regulator of gene expression. The most important p300 functions during transcriptional regulation include serving as a HAT and a platform for other transcriptional regulators to interact with each other [31, 32]. Histone acetylation by p300 has recently been found to synergize with histone methylation during transcriptional activation [33], suggesting that p300 functions may be important for histone modifications in addition to acetylation. By unknown mechanisms, cancers with a poor prognosis often contain hypoacetylated H3K18 [4]. Ad E1A 243R is capable of inducing H3K18 hypoacetylation when expressed in cells [4, 24]. Importantly, p300 or CBP appears to be the main enzyme responsible for H3K18 acetylation [4, 34].

In this report, we examined how p300 HAT functions are modulated by the Ad E1A N-terminal repression domain (E1A 1-80). Our data demonstrate strong inhibition by E1A 1-80 of p300-mediated acetylation of H3K18 in reconstituted chromatin. Since H3K18 is one of the histone lysine residues favored by p300 [23, 34], the level of H3K18 acetylation may serve as a measure of p300 function in transcription. Thus, inhibition of H3K18 acetylation by E1A 1-80 could well be involved in repression by E1A 1-80 from reconstituted chromatin [2].

A potentially important observation is that p300 autoacetylation is enhanced by E1A 1-80. Previous studies have shown that p300 autoacetylation affects its structure and HAT enzymatic activity [25, 35]. E1A 1-80 interaction with p300 may induce conformational changes in p300 so that some lysine residues become more accessible to p300 HAT activity and thus more heavily acetylated. The nature of the specific residues that E1A 1-80 targets to enhance p300 autoacetylation are not known.

How does E1A 1-80 enhance p300 autoacetylation and concurrently inhibit H3K18 acetylation? One possibility is that E1A 1-80 induces structural changes in p300 that increases p300 autoacetylation and reduces p300's ability to acetylate H3K18. This could be due to reduced p300 binding to chromatin or diminished p300 HAT activity caused by autoacetylation-induced conformational changes in p300 or by interaction with E1A 1-80. Alternatively, enhanced autoacetylation of p300 by E1A 1-80 may result in increased dissociation of p300 from the promoter [26]. Our results may suggest a mechanism for E1A 1-80-mediated transcription-repression. By enhancing p300 autoacetylation, E1A 1-80 may reduce the ability of p300 to acetylate H3K18 in chromatin, and this could result in transcriptional repression due to inhibition of nucleosome clearance from chromatin.

Thomas and Chiang [28] demonstrated that the HPV E6 oncoprotein represses p53-dependent gene activation by inhibiting p300 acetylation of p53 and nucleosomal core histones in reconstituted chromatin. Their findings indicate that a p53/E6/p300 complex is critical for repression of p53–targeted gene activation. The authors suggest that E6 acts as a “molecular switch” that converts p53/p300 from an activating complex to a “repressing entity”. Although there may be fundamental similarities between the mechanism of E6 and E1A 1-80 repression, there are significant differences. For example, unlike E1A 1-80, E6 does not affect p300 autoacetylation [28].

We previously identified TFIID as well as p300 as important targets for E1A transcriptional repression and proposed a model in which a p300/E1A 1-80 complex bound upstream of the promoter causes dissociation of TFIID from the TATA box [16]. The results described here are not inconsistent with this model. It is possible that the E1A N-terminal repression domain enhances autoacetylation of promoter-bound p300, leading to inhibition of downstream H3K18 acetylation as well as promoting TFIID dissociation from the promoter [6].

It has been reported that autoacetylated p300 dissociates more efficiently from the promoter in reconstituted chromatin thus facilitating recruitment of TFIID and subsequent PIC formation resulting in transcription [26]. Perhaps E1A 1-80, in a complex with enhanced autoacetylated p300, can bind to TFIID and dissociate it from the promoter thus disrupting PIC formation. This potential complex is not unlike the p53/E6/p300 complex indicated by Thomas and Chiang, and discussed above (27). Our present results combined with our previous findings suggest that E1A can repress transcription by multiple strategies including altering chromatin-modifying activities of p300 and dissociating TFIID from the TATA box thus disrupting formation of the pre-initiation complex [5, 6].

p300 autoacetylation and HAT activity in vivo may be dynamically regulated by various factors and play important roles in gene regulation. For example, targeted down-regulation of the histone deacetylase SIRT2 with shRNA resulted in hyperacetylated p300 which was less capable of activating a p300-responsive promoter in vivo [36]. The enhancement of p300 autoacetylation and inhibition of p300-mediated histone acetylation may prove to be critical for the transcriptional repression function of the E1A N-terminal repression domain.

## MATERIALS AND METHODS

### Adenovirus infection

Adenovirus vectors expressing E1A 1-80 or lacZ were purified by CsCl ultra-centrifugation as described [1, 37] and used to infect HeLa cells in 6-well plates at 10 pfu/cell for 24 h.

### Chromatin assembly

In order to assemble chromatin sufficient for one in vitro acetylation assay, 63.5 ng of the in vitro transcription template pG5MLT was combined with 49 ng of core histone, as well as histone chaperones and chromatin remodeling factors in a total volume of 6 μl essentially as described [2], except that the final incubation was at 260C for 16 h. The quality of the reconstituted chromatin was examined by micrococcal nuclease (MN) digestion and agarose gel electrophoresis, followed by imaging on a ChemiDocXRS scanner (Bio-Rad, Hercules, CA) [2].

### In vitro acetylation assays and Western Blots

Purified p300, Gal4-VP16 and E1A 1-80 were combined on ice in 14 μl. Expression and purification of the recombinant proteins were described previously [2, 28]. After incubation on ice for 15 min, 6 μl of chromatin was added, and acetylation reaction carried out at 320C for 10 min. Typically, 12 nM of p300 and 264 nM of E1A 1-80 were used for in vitro acetylation assays, unless indicated otherwise. Reactions contained 20 mM Tris-HCl (pH 8.2), 0.2 mM DTT, 20 mM KCl, 2 mM Na Butyrate, 0.01mg/ml BSA, 5% glycerol, and 25 μM acetyl-CoA. Reactions were stopped with 30 μl of SDS sample loading buffer and reaction products electrophoresed on 4-12% SDS-PAGE gels. Separated proteins were transferred to PVDF-P membranes, membranes blocked with 3% milk/1xPBST (with 0.05% Tween-20) for 30 min, and incubated with primary antibody in the same buffer for 1 h. After washing for 5 min with 1xPBST, the membranes were incubated with an HRP-conjugated secondary antibody in 3% milk/1xPBST for 45 min. Membranes were then washed for 30 min with 1xPBST, briefly rinsed with water, developed with the Lumi-Light reagent (Roche), and exposed to X-ray film (Figure 1C) or scanned on Bio-Rad scanner for quantitation. Antibodies used for Western blots: p300: #SC-584x (Santa Cruz Biotech); acetylated p300: anti-acetyl-Lys, #9681 (Cell Signaling Technology); H3K18Ac: #07-354 (Millipore); H3K9Ac: #39585 (Active Motif); E1A 1-80: rabbit polyclonal antibody against E1A 1-80.
